# The ETNK2 gene promotes progression of papillary thyroid carcinoma through the HIPPO pathway

**DOI:** 10.7150/jca.65587

**Published:** 2022-01-01

**Authors:** Danni Zheng, Lingli Jin, Buran Chen, Yufeng Qi, Adheesh Bhandari, Jialiang Wen, Bangyi Lin, Xiaohua Zhang, Wei Zhang

**Affiliations:** 1 Department of Breast Surgery, The First Affiliated Hospital of Wenzhou Medical University, Wenzhou, Zhejiang, PR China.; 2 Department of Surgery, Breast and Thyroid Unit, Primera Hospital, Kathmandu, Nepal.

**Keywords:** ETNK2, PTC, Hippo, EMT

## Abstract

Thyroid cancer is a disease with an extremely high incidence rate and is divided into papillary, follicular, medullary, and undifferentiated thyroid cancers. Among them, papillary carcinoma is the most common subtype. We assessed expression of ETNK2 in public databases and found that ETNK2 is upregulated in PTC. Cohort and RNA sequencing data were used to verify this discovery. To further determine the relationship between ETNK2 and papillary thyroid carcinoma, we performed an in vitro experiment. In a PTC cell line, silencing ETNK2 inhibited cell proliferation, weakened cell migration and invasion ability, promoted apoptosis, and blocked the cell cycle. In addition, western blotting suggested that ETNK2 is related to the HIPPO pathway and may activate the EMT pathway through the HIPPO pathway to promote the development of thyroid cancer. These results revealed that ETNK2 is related to the occurrence and development of papillary thyroid carcinoma, suggesting that ETNK2 may be an oncogene associated with PTC.

## Introduction

With the improvement of diagnostic technology, the incidence of thyroid cancer (TC) has increased significantly 1-5, and papillary thyroid carcinoma (PTC) is the most common pathological subtype of thyroid cancer 2, 6-9. The prognosis of PTC patients is usually relatively good 7. Surgical removal of the lesion is the most important method to treat papillary thyroid carcinoma, followed by I^131^ treatment 7, 9, 10. Long-term survival after surgical treatment or radioactive iodine treatment is 90-95% effective 9; however, patients who experience local infiltration or distant recurrence may have a greatly increased risk of death 10. Therefore, understanding the disease development process and identifying early diagnostic indicators are key to improving the prognosis of PTC patients.

Elevations in phosphoethanolamine (PE) are often observed in human cancers and xenografts 11, 12. ETNK2 is one of the enzymes involved in the CDP-ethanolamine pathway in mammals, converting ethanolamine into phosphoethanolamine 13, 14. Expression of ETNK-2 in the liver and reproductive tissues exceeds that in other organs 13. HCC with higher ETNK2 mRNA levels may retain characteristics of hepatocytes and has a higher degree of differentiation than HCC with low expression. Patients with high ETNK2 mRNA expression levels exhibit increased survival. It has been suggested that mRNA levels of ETNK2 may predict survival after HCC diagnosis 15. Studies have shown that upregulation of ETNK2 in GC enhances liver metastasis. This may be related to the dysregulation of p53-Bcl-2-related apoptosis 16. Studies have shown that methylation of the ETNK2 gene promoter helps laryngeal cancer cells resist radiation 17. ETNK2 expression is significantly upregulated in lung adenocarcinoma and squamous cell carcinoma 18. After silencing ETNK2 in MDA-MB-231 cells, cell viability was significantly reduced 19. ETNK2 is also involved in haemostasis. Due to extensive placental thrombosis, ETNK2-/- mice usually do not survive foetal development and succumb during the second trimester of pregnancy, indicating a key role of the ETNK2 sequence in haemostasis 13. ETNK2 has been identified as a new molecular marker for the BRCA site 20. ETNK2 suggests the significance of survival risk in some cancers and serves as a new molecular marker, but its role in PTC has not yet been elucidated.

In this study, we examined expression of ETNK2 in PTC using the TCGA database and our validation data. We also analysed the relationship between ETNK2 expression and the clinicopathological characteristics of PTC. Finally, we knocked out ETNK2 and conducted functional analysis and WB experiments to explore the role that ETNK2 plays in PTC. Moreover, we demonstrated that ETNK2 is highly expressed in PTC cell lines. In an in vitro study, ETNK2 promoted cell proliferation, colony formation, migration, and invasion, while inhibiting apoptosis. These phenotypes are associated with two pathways described below.

Epithelial-mesenchymal transition (EMT) is a cellular process that enables epithelial cell transformation toward the mesenchymal phenotype, which results in metastasis and invasion in cancer 21-23. During the EMT process, interstitial genes such as N-cadherin and vimentin are activated, while the expression of E-cadherin decreases 24. During this process, epithelial cells lose cell polarity, lose their connection with the basement membrane and other epithelial phenotypes, and acquire mesenchymal phenotypes such as high migration and invasion, anti-apoptosis, and the ability to degrade extracellular matrix 22.

The Hippo signalling pathway plays a crucial role in regulating tumour cell proliferation and apoptosis 25. YAP and TAZ are key proteins in the HIPPO pathway that are overexpressed in many tumours 26. A large amount of in vitro evidence shows that YAP and/or TAZ activation promotes proliferation and proliferation. Recently, some studies have clarified that the HIPPO signalling pathway can regulate EMT to promote tumour invasion and metastasis 25-27. Overall, these studies show that in cancer cells, increased YAP or TAZ expression or their activation can disrupt cell-to-cell connections, promote mesenchymal gene expression, and enhance EMT-related morphological changes. Otherwise, EMT is suppressed.

In conclusion, this work aimed to determine the relationship between ETNK2 expression and its role in thyroid cancer proliferation, migration, invasion, apoptosis, and cell cycle arrest.

## Materials and Methods

### Patients and samples

We obtained the clinical data of ETNK2 and its expression in different samples from the TCGA database. We obtained surgical specimens and control tissues adjacent to cancer and clinical data from 40 patients diagnosed with papillary thyroid carcinoma in the First Affiliated Hospital of Wenzhou Medical University over the past two years. The primary case inclusion criteria were as follows: (1) patients whose primary tumour was thyroid cancer with no other organs severely affected; (2) patients who had undergone total or near total thyroidectomy and had not received radiotherapy; (3) patients with no medical history of other malignant tumour; and (4) patients with complete clinical baseline characteristics. The primary case exclusion criteria were as follows: (1) patients with other malignancies or a history of other malignancies; (2) patients suffering from serious illness such as heart failure, stroke or chronic renal failure; and (3) patients with a history of I^131^ treatment. This study obtained informed consent from patients with the approval of the Ethics Committee of the First Affiliated Hospital of Wenzhou Medical University.

### Cell lines and cell culture

Professor Mingzhao Xing of Johns Hopkins University School of Medicine in Baltimore, Maryland, US provided us with human thyroid cancer cell lines (BCPAP, TPC-1, and KTC-1). We purchased a control thyroid cell line (HTORI3) from the Chinese Academy of Sciences. Cells were cultured in 1640 medium (Invitrogen, Carlsbad, CA, USA) containing 10% foetal bovine serum (FBS; Invitrogen) and cultured in an incubator containing 5% carbon dioxide at 37°C.

### Cell transfection

We entrusted Gene Pharma (Shanghai, China) to design the siRNAs. Before transfection, we inoculated cells into six-well plates (TPC-1, BCPAP: 75,000 cells per well). Our transfection ratio was 7.5 µl SI per 3 µl Lipofectamine RNAiMAX (Invitrogen, Grand Island, NY, USA). We collected the cells 48 h after transfection for the next experiment. The siRNA sequences of ETNK2 were as follows: Sense‐1: GCACAAUUAUUUCACGCUUTT; antisense‐1: AAGCGUGAAAUAAUUGUGCTT; Sense‐2: GGUACGCAGUGAUCCGAUUTT; antisense‐2: AAUCGGAUCACUGCGUACCTT.

### Quantitative real-time polymerase chain reaction (qRT-PCR)

We used a triazole reagent (Invitrogen; Thermo Fisher Scientific, Inc.) to lyse the tissue and extract total RNA to measure RNA concentration. Then, the reverse transcription reagent ReverTra Ace qPCR RT Kit (Toyobo Life Science) was used to reverse transcribe 1 μg of RNA per sample. SYBR Green Real-time PCR Master Mix (TOYOBO, QPK-201-201T) was used to amplify cDNA. The PCR instrument used was an ABI 7500 Fast Real-Time PCR System (Applied Biosystems; Thermo Fisher Scientific Inc.). The sequences of the primers used were as follows: ETNK2 sense: GAAGGAGCATCTGTCCCAGC; ETNK2 antisense: CAATGAACCGCACGTGACCT; GAPDH sense: 5′-GGTCGGAGTCAACGGATTTG-3′, GAPDH antisense: 5′-ATGAGCCCCAGCCTTCTCCAT-3′.

### Cell proliferation assays

CCK-8 and cell cloning experiments were used to determine cell proliferation. We inoculated the transfected cells into 96-well plates at 1000 cells per well for TPC-1 and BCPAP cells, incubated them with CCK-8 reagent for 3 hours, and then measured the absorbance for 4 consecutive days. In the cell cloning experiment, we seeded the same number of cells into 6-well plates, fixed the cells in 4% paraformaldehyde 6 days later, and stained them with crystal violet. The above experiment was repeated 3 times.

### Migration and invasion experiment

Cells were transfected for 48 hours and inoculated into the upper chamber of a Transwell plate with serum-free medium (40,000 cells per well), and the total volume of the upper chamber was 300 µl. Next, 600 µl medium containing 10% foetal bovine serum was added to the lower chamber. After culturing for 22 hours, the culture medium was discarded, a cotton swab was used to remove nonmigrated cells, the migrated cells on the lower surface were fixed, and the cells were stained with 0.01% crystal violet for 30 minutes. The cell invasion experiment was performed in the same way, and the culture time was extended to 24 h. The above experiment was performed at least 3 times.

### Apoptosis assays

An apoptosis kit (#556547; Becton, Dickinson and Company, Franklin Lakes, NJ, USA) was used to assess apoptosis. The transfected cells were collected and washed 3 times with PBS. Then, the cells were resuspended in 500 µl of 1× binding buffer. Then, the cells were stained with Annexin V-FITC for 15 min and PI for 5 min while protected from light. Flow cytometry was used to measure apoptosis, and FlowJo software was used to analyse the results.

### Cell cycle assays

7-Amino-actinomycin D (7-AAD, #559925, Becton, Dickinson, and Company) was used to determine cell cycle distribution. The transfected cells were collected and washed 3 times with PBS. Thyroid cells were cultured in serum-free medium before transfection and starved for 24 hours. Forty-eight hours after transfection, the cells were fixed in 75% ethanol and placed in a refrigerator at -20℃ overnight. Next, cells were washed and resuspended in PBS followed by staining with 5 µl 7-AAD189 solution for 10 min in the dark. Finally, samples were run on the streaming machine. The results were analysed using Modfit LT 5.0 software (Verity Software House, Topsham, ME).

### Western blot experiments

Cells were collected 48 h after transfection and lysed for quantitative denaturation. Proteins were electrophoresed on 10% SDS-PAGE, with 20 µg of protein per well. Then, the gels were transferred to polyvinylidene fluoride (ISEQ00010, microporous) membranes via electrophoresis. After electrophoresis was complete, 1× fast blocking solution was used to block the membranes for 10-15 minutes, followed by washing with PBS three times and incubation overnight at 4°C with primary antibodies. After washing 3 times with TBST, the membranes were incubated with anti-mouse IgG and anti-rabbit IgG secondary antibodies (Abcam, Cambridge, MA) for 1 h at room temperature. The intensity of all bands was quantified using Image Laboratory software.

## Results

### Expression of ETNK2 is significantly upregulated in PTC

**Figure 1:**
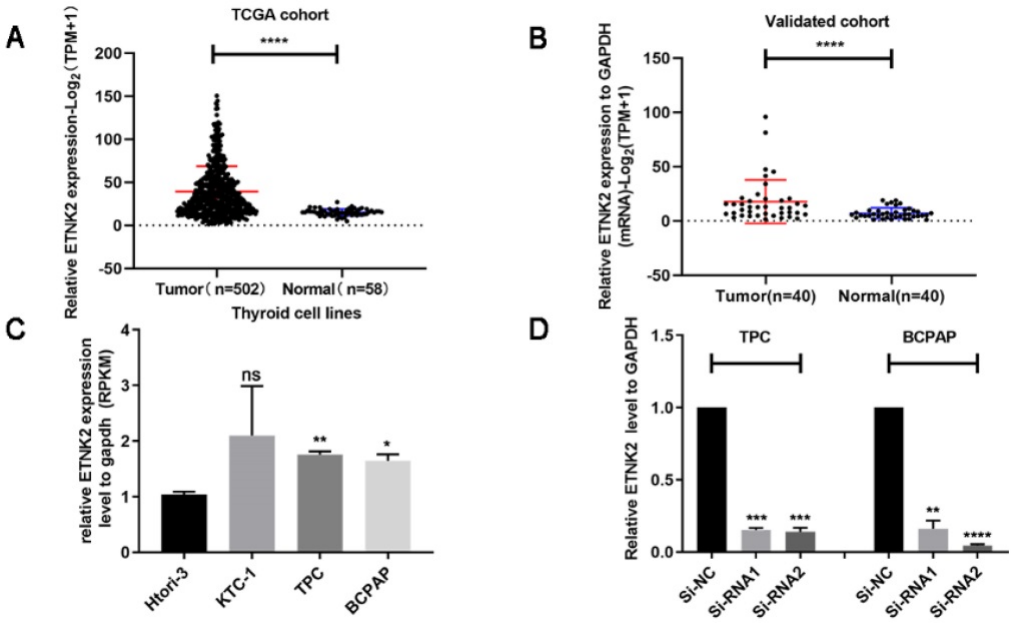
ETNK2 was overexpressed in PTC. (A) Levels of ETNK2 mRNA in PTC tissue in TCGA were higher than that in control thyroid tissue. (B) Using qRT-PCR, we verified overexpression of ETNK2 in PTC. (C) Compared to the control cell line HTORI-3, ETNK2 is upregulated in PTC cell lines (TPC-1 and BCPAP). (D) In both cell lines, expression levels of Si-RNA1 and Si-RNA2 were lower than that of Si-NC. Statistical analyses were performed as follows: (A): Mann-Whitney test; (B): Wilcoxon test; (C, D): Student's t-test; *p<0.05, **p<0.01, ****p<0.0001.

We analysed the TCGA database to explore the function of ETNK2 in PTC and found that expression of ETNK2 in PTC was significantly increased compared to that in nonneoplastic thyroid tissue (Figure 1A). Similarly, we also detected mRNA expression levels of ETNK2 in 40 patients by RT-qPCR. This result was consistent with that of TCGA validation (Figure 1B).

### Overexpression of ETNK2 is related to the clinicopathologic features of PTC

To further explore the effect of ETNK2 in PTC, we evaluated the correlation between expression levels of ETNK2 in the TCGA database and our validation cohort and the clinical characteristics of PTC. According to the different levels of ETNK2 expression, we divided patients into groups with high expression of ETNK2 (n=251) and low expression of ETNK2 (n=251). As shown in Table 1, histological type (P<0.001), lymph node metastasis (P<0.001), and disease stage (P=0.011) were associated with high expression of ETNK2. In contrast, sex, age, T stage, and distant metastasis did not correlate with the expression level of ETNK2. In the same way, we divided the validation cohort into a high expression group (n=20) and a low expression group (n=20) according to the level of expression (Table 2). Expression of ETNK2 was not related to the above clinicopathological characteristics, possibly due to the small sample size. Taken together, these results suggest that ETNK2 may play an oncogenic role in PTC.

### High expression of ETNK2 may lead to multifocal tumours in PTC patients

We performed univariate logistic regression analysis on TCGA data. Results suggested that high ETNK2 expression (odds ratio [OR]=1.539, 95% confidence interval [CI]: 1.013-2.338, p=0.043), <45 years old (OR=0.032, CI=0.010-0.105, P<0.001), classical type (OR=2.81, CI=1.732-4.557, P<0.001), and disease stage III/IV (OR=59.953, CI=17.291-207.880, P<0.001) were related to LNM. The multivariate logistic regression analysis indicated that ETNK2 expression (OR=1.536, CI: 1.014-2.329, p=0.043), <45 years old (OR=0.031, CI=0.009-0.100, P<0.001), classical type (OR=2.804, CI=1.739-4.520, P<0.001), and disease stage III/IV (OR=66.615, CI=20.101-220.761, P<0.001) were independent risk factors for LNM. These results indicated that high expression levels of ETNK2 are a high-risk factor for lymph node metastasis in PTC.

### ETNK2 promotes proliferation and colony formation of PTC cells

We assessed mRNA levels of ETNK2 in several PTC and normal thyroid cell lines and found that expression of ETNK2 in TPC-1 and BCPAP cells was significantly higher than that in control HTORI-3 cells (Figure 1C). Using small interfering RNA (siRNA) to knockdown TPC-1 and BCPAP significantly reduced the levels of ETNK2 mRNA (Figure 1D).

**Figure 2:**
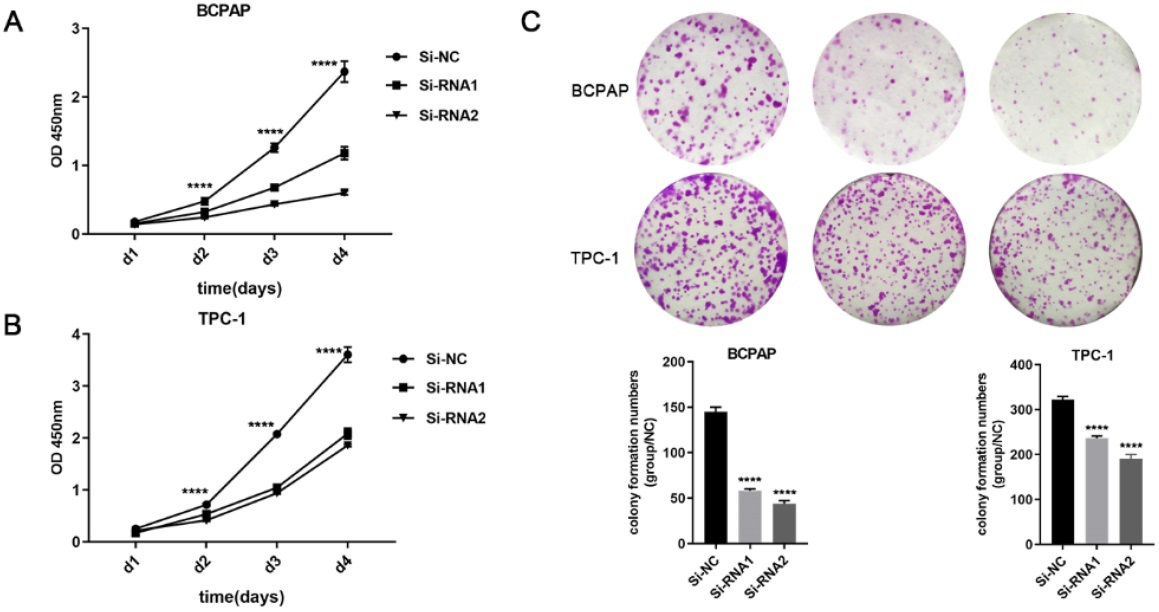
ETNK2 promotes proliferation and colony formation in PTC cells. (A, B) CCK8 assays in PTC cells. (C) colony formation assays and colony numbers. The data were analysed by Student's t test.

Next, CCK-8 and clone formation experiments were performed to explore the effect of ETNK2 on the proliferation of PTC cells. As shown in Figure 2A-C, CCK-8 and colony formation experiments revealed that downregulation of ETNK2 significantly inhibited the proliferation of TPC-1 and BCPAP cells.

### Downregulation of ETNK2 gene expression decreases the migration and invasion ability of PTC cells

**Figure 3:**
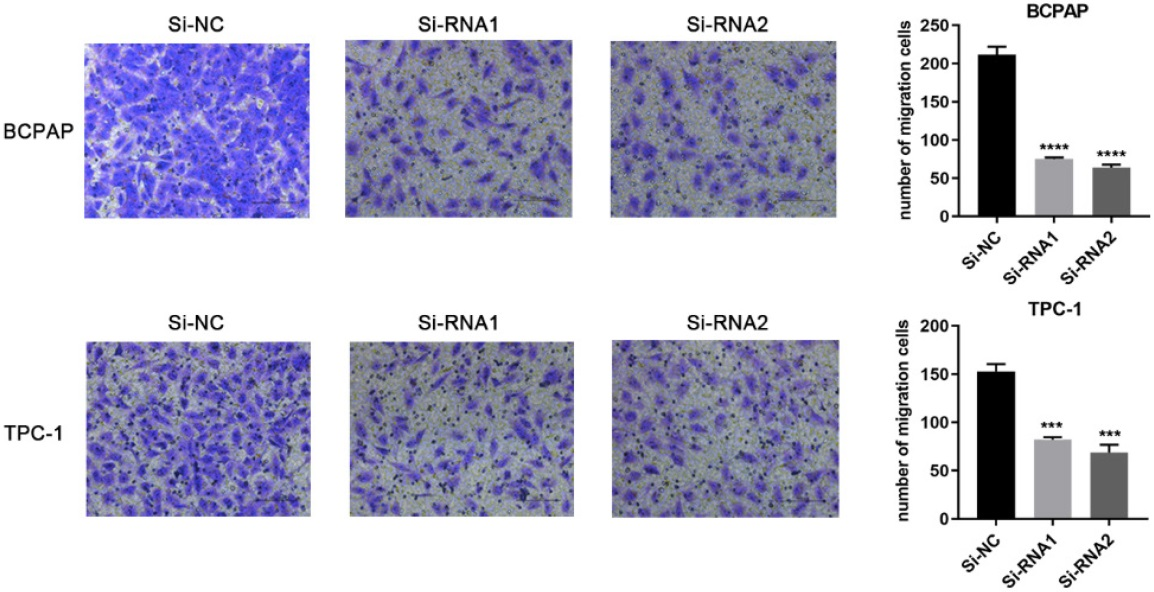
Downregulation of ETNK2 inhibits the migration of PTC cell lines.

**Figure 4:**
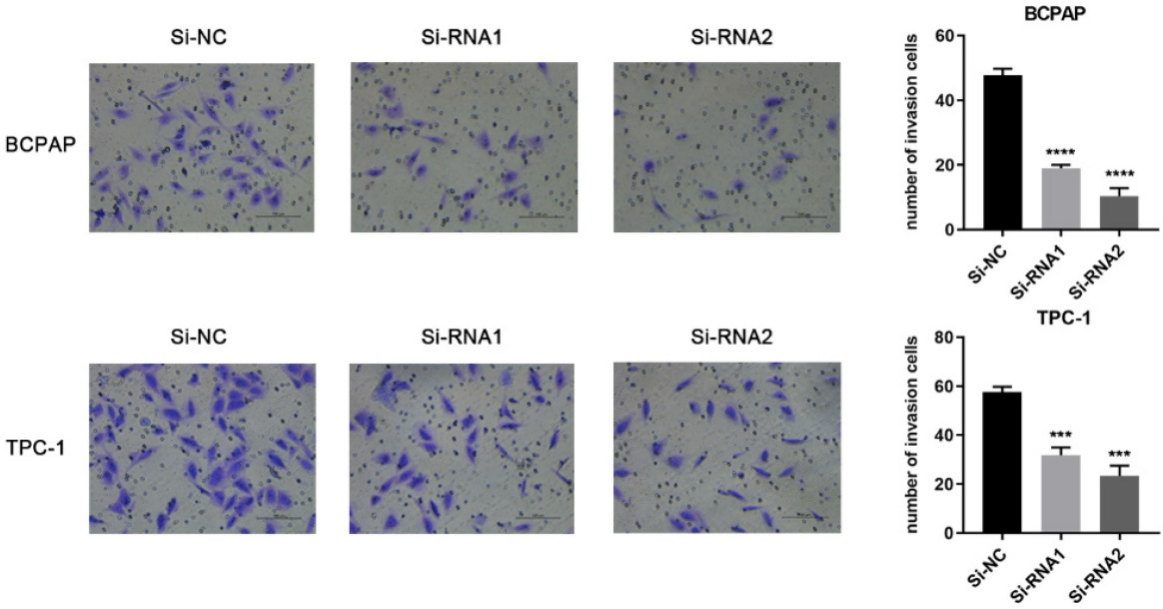
Downregulation of ETNK2 inhibits the invasion of PTC cell lines.

Based on the CCK-8 assay and colony formation assay, we speculated that ETNK2 may be related to the migration and invasion of PTC cells. Compared to Si-NC, the ability of the knockdown group to migrate migration and invasion was inhibited (Figure 3). This phenomenon was also observed in the invasion experiment (Figure 4).

### Low ETNK2 expression in PTC is related to decreased apoptosis

**Figure 5:**
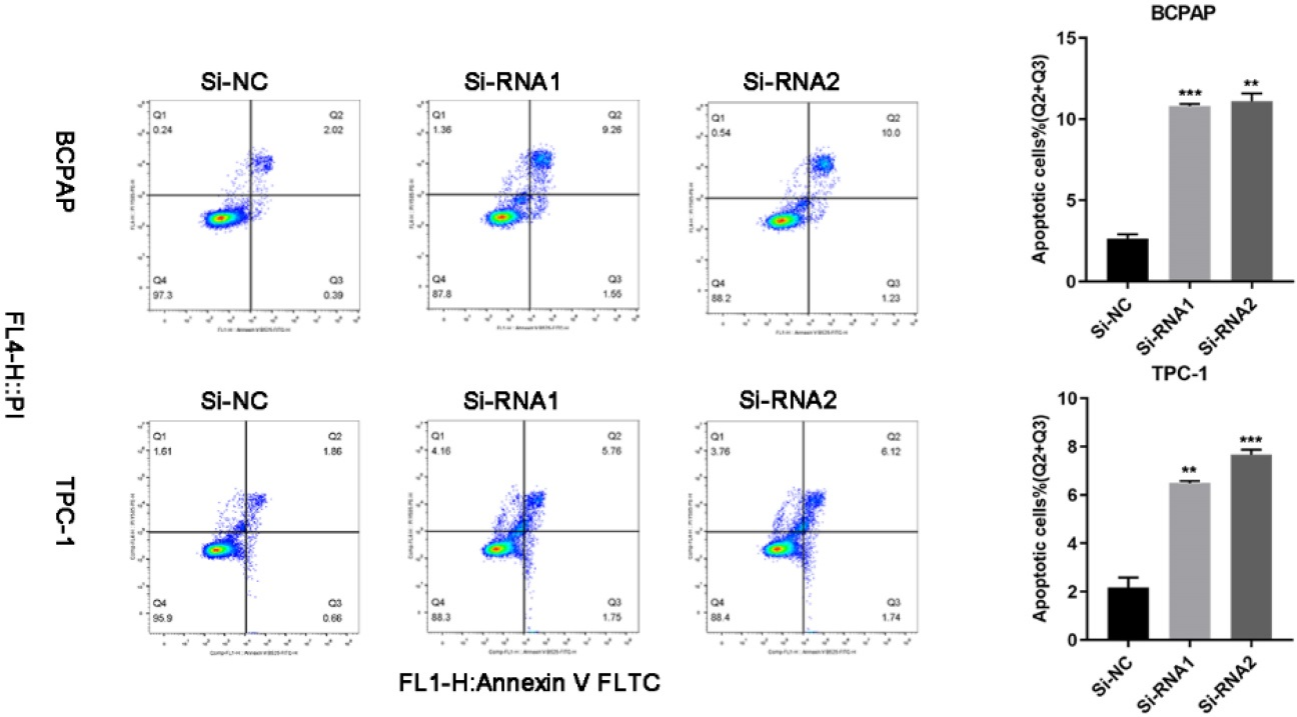
Silencing ETNK2 promotes apoptosis.

We used flow cytometry to analyse the cells and found that the number of cells undergoing apoptosis in the early and late stages of transfection was increased. Q2 (early apoptosis) plus Q3 (late apoptosis) is the total cellular apoptosis rate. The apoptosis rate after transfection was increased (Figure 5).

### Downregulation of ETNK2 can cause cycle arrest

**Figure 6:**
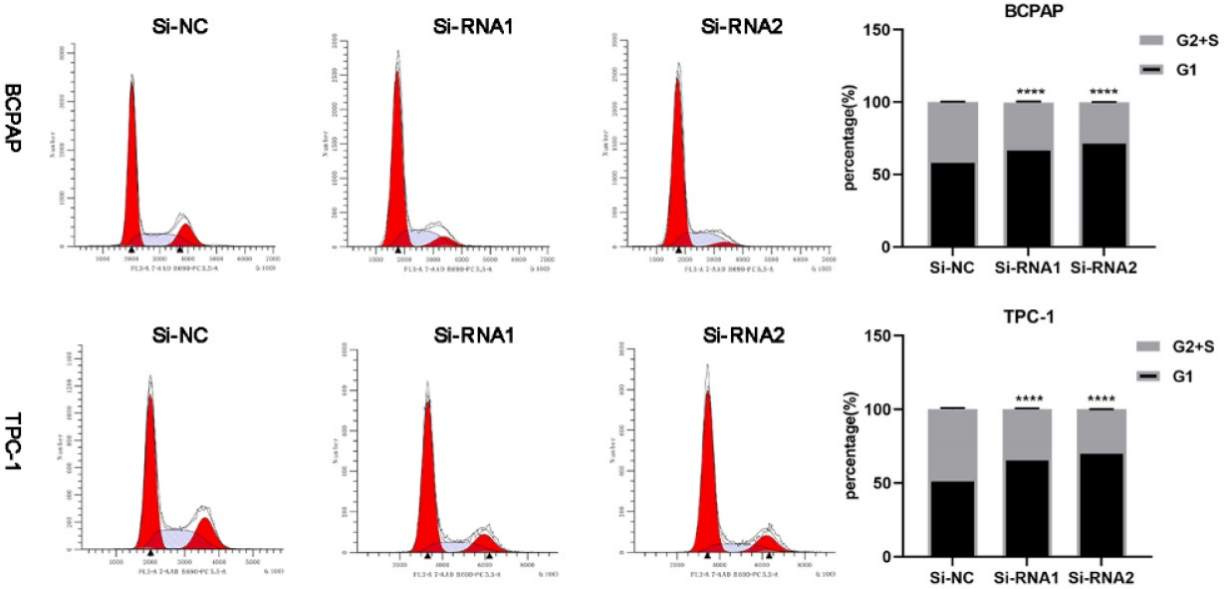
Silencing ETNK2 blocks the cell cycle.

**Figure 7:**
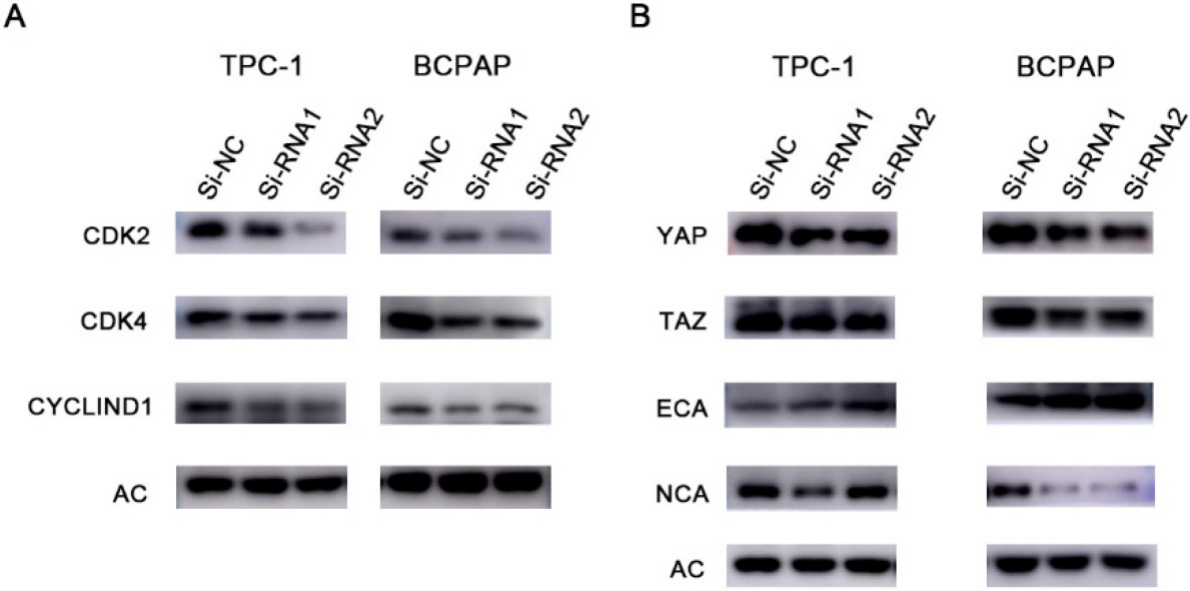
Protein changes in response to silencing ETNK2. (A) Cyclin changes at the protein level after silencing ETNK2, including downregulation of CDK2, CDK4, and CYCLIND1. (B) Silencing ETNK2 activates the HIPPO and EMT pathways. YAP and TAZ are downregulated in HIPPO, N-cadherin is downregulated in EMT, and E-cadherin is upregulated.

To clarify the effect of knockdown on the cell cycle and cell cycle distribution, we analysed transfected cells using flow cytometry. As shown in Figure 6, the number of treated cells resting in G1 phase increased, and ETNK2 knockdown resulted in G1/S phase arrest. In the WB experiment, expression levels of CDK2, CDK4, and CyclinD1 in the knockdown group were also significantly lower than those in the control group (Figure 7A).

### ETNK2 may promote the development of thyroid cancer through the HIPPO and EMT pathways

We next verified the role of ETNK2 at the protein level. WB experiments showed that expression levels of YAP, TAZ, and NCA in the knockout group were decreased, while expression levels of ECA were increased. These results suggest that ETNK2 may play a role in promoting cancer through the HIPPO and EMT pathways (Figure 7B).

## Discussion

With changes in follow-up treatment and living habits, the incidence of thyroid cancer is increasing each year, and thyroid cancer currently is the sixth most common cancer in women. Although its prognosis is good, many people still die from the disease each year. Therefore, identifying high-risk patients is a major challenge that needs to be solved 28, 29.

Elevated phosphoethanolamine levels are often observed in some cancers and allografts 11, 12. We suspect that elevated PE may have a relationship with the occurrence of cancer. Since ETNK2 ginseng is one of the mammalian enzymes involved in the CDP-ethanolamine pathway, it plays a role in converting ethanolamine into phosphoethanolamine 13, 14, so we consulted the relevant literature. Under normal circumstances, ETNK2 is highly expressed in the liver and reproductive tissues 13. Some studies have confirmed that increased expression of ETNK2 is closely related to liver metastasis in gastric cancer 16, lung adenocarcinoma 18, and lung squamous cell carcinoma 18. At present, there is no literature concerning the relationship between ETNK2 and thyroid malignant tumours, so we investigated this relationship to explore new molecular markers of PTC.

In our study, we found for the first time in a public database that expression of ETNK2 is highly upregulated in papillary thyroid carcinoma compared to control thyroid tissue. Similarly, we used 40 of our tissues, including cancerous tissues and para-cancerous tissues, to conduct mRNA analysis and obtained the same results. We investigated the relationship between expression of ETNK2 in PTC and clinical case characteristics. Patients with high levels of ETNK2 exhibited a higher incidence of the classical type and were more prone to lymph node metastasis and later disease staging. Regression analysis indicated that the high expression of ETNK2 was closely related to lymph node metastasis. ETNK2 overexpression was an independent high-risk predictor of PTC lymph node metastasis. These results indicated that ETNK2 may represent a potential valuable molecular marker of PTC.

In our cell function experiments, we confirmed that ETNK2 promotes the clonal proliferation, migration, proliferation, apoptosis, and cell cycle arrest of papillary thyroid carcinoma. In the WB experiment, after knocking down ETNK2, we found that YAP, TAZ, N-cadherin, and other proteins were downregulated, and E-cadherin was upregulated. Many studies have found that the HIPPO and EMT pathways may play an interactive role in the occurrence and development of cancer. We speculate that in PTC, ETNK2 may activate EMT through the HIPPO pathway, promoting tumour migration and proliferation.

In summary, the biological role of ETNK2 in PTC suggests that it may be a potential molecular marker and is expected to become a new therapeutic target.

## Figures and Tables

**Table 1:** The association between ETNK2 expression and clinicopathologic features in the TCGA cohort.

Clinicopathologic factors	Patients	High expression	Low expression	p-value
Gender				
Female	367	180	187	
Male	135	71	64	0.481
Age(years)				
<45	236	116	120	
≥45	266	135	131	0.721
Histological type				
Classical	356	201	155	
Other types	146	50	96	<0.001*
T stage				
Ⅰ+Ⅱ	309	144	163	
Ⅲ+Ⅳ	193	106	87	0.081
Lymph node metastasis				
No	280	117	158	
Yes	222	128	89	<0.001*
Disease stage(AJCC7)				
Ⅰ+Ⅱ	335	154	181	
Ⅲ+Ⅳ	167	97	70	0.011*
Metastasis				
No	493	246	247	
Yes	9	5	4	0.737

* P<0.05

**Table 2:** The correlation between ETNK2 expression and clinicopathologic factors in the validated cohort.

Clinicopathologic factors	Patients	High expression	Low expression	p-value
Gender				
Female	26	13	13	
Male	14	7	7	1.000
Age(years)				
<45	26	13	13	
≥45	14	7	7	1.000
T stage				
Ⅰ+Ⅱ	19	9	10	
Ⅲ+Ⅳ	21	11	10	0.752
Lymph node metastasis				
No	18	10	8	
Yes	22	10	12	0.525
Disease stage(AJCC7)				
Ⅰ+Ⅱ	34	18	16	
Ⅲ+Ⅳ	6	2	4	0.376

**Table 3:** Univariate and multivariate COX regression analysis for overall survival in the TCGA cohort.

Clinicopathologic factors	Univariate analysis			Multivariate analysis		
	OR	95% CI	P-value	OR	95% CI	P-value
ETNK2 Expression (high vs. low)	1.539	1.013-2.338	0.043*	1.536	1.014-2.329	0.043*
Gender (female vs. male)	0.701	0.437-1.126	0.142		-	
Age (>45 vs. <45)	0.032	0.010-0.105	<0.001*	0.031	0.009-0.100	<0.001*
Metastasis (yes vs. no)	0.646	0.155-2.685	0.548		-	
Histological type (classical vs. others)	2.810	1.732-4.557	<0.001*	2.804	1.739-4.520	<0.001*
T stage (III/IV vs. I/II)	1.130	0.704-1.816	0.613		-	
Disease stage (AJCC7) (III/IV vs. I/II)	59.953	17.291-207.880	<0.001*	66.615	20.101-220.761	<0.001*

* P<0.05

## References

[1] Seib C, Sosa JJE, America mcoN (2019). Evolving Understanding of the Epidemiology of Thyroid Cancer. Endocrinol Metab Clin North Am.

[2] Schneider D, Chen HJCacjfc (2013). New developments in the diagnosis and treatment of thyroid cancer. CA Cancer J Clin.

[3] Lee J, Shin SJL (2014). Overdiagnosis and screening for thyroid cancer in Korea. Lancet.

[4] Kitahara C, Sosa J (2020). Understanding the ever-changing incidence of thyroid cancer. Nat Rev Endocrinol.

[5] Dong X, Song J, Hu J, Zheng C, Zhang X, Liu HJFic BRAFT-Box Transcription Factor 22 Is an Immune Microenvironment-Related Biomarker Associated With the Mutation in Papillary Thyroid Carcinoma. 2020; 8: 590898.

[6] Burns W, Zeiger MJSio (2010). Differentiated thyroid cancer. Semin Oncol.

[7] Cabanillas M, McFadden D, Durante CJL (2016). Thyroid cancer. Lancet.

[8] Differentiated thyroid cancer (2013). getting the complete picture. %J Lancet (London, England). Lancet.

[9] Pacini F, Castagna M, Brilli L, ESMO Guidelines Working Group (2009). Differentiated thyroid cancer: ESMO clinical recommendations for diagnosis, treatment and follow-up. Ann Oncol.

[10] Sherman S (2003). Thyroid carcinoma. Lancet.

[11] Podo F (1999). Tumour phospholipid metabolism. NMR Biomed.

[12] Negendank W (1992). Studies of human tumors by MRS: a review. NMR Biomed.

[13] Tian Y, Jackson P, Gunter C, Wang J, Rock C, Jackowski S (2006). Placental thrombosis and spontaneous fetal death in mice deficient in ethanolamine kinase 2. J Biol Chem.

[14] Lykidis A, Wang J, Karim M, Jackowski S (2001). Overexpression of a mammalian ethanolamine-specific kinase accelerates the CDP-ethanolamine pathway. J Biol Chem.

[15] Guan Y, Chen X, Wu M, Zhu W, Arslan A, Takeda S (2020). The phosphatidylethanolamine biosynthesis pathway provides a new target for cancer chemotherapy. J Hepatol.

[16] Miwa T, Kanda M, Shimizu D, Umeda S, Sawaki K, Tanaka H (2021). Hepatic metastasis of gastric cancer is associated with enhanced expression of ethanolamine kinase 2 via the p53-Bcl-2 intrinsic apoptosis pathway. British Journal of Cancer.

[17] Kim JS, Kim SY, Lee M, Kim SH, Kim SM, Kim EJ (2015). Radioresistance in a human laryngeal squamous cell carcinoma cell line is associated with DNA methylation changes and topoisomerase II alpha. Cancer Biol Ther.

[18] Lesko J, Triebl A, Stacher-Priehse E, Fink-Neuböck N, Lindenmann J, Smolle-Jüttner F (2021). Phospholipid dynamics in ex vivo lung cancer and normal lung explants. Exp Mol Med.

[19] Shah T, Krishnamachary B, Wildes F, Wijnen J, Glunde K, Bhujwalla Z (2018). Molecular causes of elevated phosphoethanolamine in breast and pancreatic cancer cells. NMR Biomed.

[20] Alshabi A, Shaikh I, Vastrad C (2019). Exploring the Molecular Mechanism of the Drug-Treated Breast Cancer Based on Gene Expression Microarray. Biomolecules.

[21] Lu W, Kang Y (2019). Epithelial-Mesenchymal Plasticity in Cancer Progression and Metastasis. Dev Cell.

[22] Nieto M, Huang R, Jackson R, Thiery J (2016). EMT: 2016. Cell.

[23] Chaffer C, San Juan B, Lim E, Weinberg R (2016). EMT, cell plasticity and metastasis. Cancer Metastasis Rev.

[24] Lamouille S, Xu J, Derynck R (2014). Molecular mechanisms of epithelial-mesenchymal transition. Nat Rev Mol Cell Biol.

[25] Pan D (2010). The hippo signaling pathway in development and cancer. Dev Cell.

[26] Warren J, Xiao Y, Lamar J (2018). YAP/TAZ Activation as a Target for Treating Metastatic Cancer. Cancers (Basel).

[27] Zanconato F, Cordenonsi M, Piccolo S (2016). YAP/TAZ at the Roots of Cancer. Cancer Cell.

[28] Bhandari A, Guan Y, Xia E, Huang Q, Chen Y (2019). VASN promotes YAP/TAZ and EMT pathway in thyroid carcinogenesis in vitro. Am J Transl Res.

[29] Dong X, Yang Q, Gu J, Lv S, Song D, Chen D Identification and validation of L Antigen Family Member 3 as an immune-related biomarker associated with the progression of papillary thyroid cancer. 2021; 90: 107267.

